# The novel phospholipid mimetic KPC34 is highly active against preclinical models of Philadelphia chromosome positive acute lymphoblastic leukemia

**DOI:** 10.1371/journal.pone.0179798

**Published:** 2017-06-23

**Authors:** Peter M. Alexander, David L. Caudell, Gregory L. Kucera, Kristin M. Pladna, Timothy S. Pardee

**Affiliations:** 1 Internal Medicine, Section on Hematology and Oncology, Wake Forest Baptist Health, Winston-Salem, North Carolina, United States of America; 2 Pathology-Comparative Medicine, Wake Forest Baptist Health, Winston-Salem, North Carolina, United States of America; 3 Cancer Biology, Comprehensive Cancer Center of Wake Forest University, Winston-Salem, North Carolina, United States of America; European Institute of Oncology, ITALY

## Abstract

Philadelphia chromosome positive B cell acute lymphoblastic leukemia (Ph+ ALL) is an aggressive cancer of the bone marrow. The addition of tyrosine kinase inhibitors (TKIs) has improved outcomes but many patients still suffer relapse and novel therapeutic agents are needed. KPC34 is an orally available, novel phospholipid conjugate of gemcitabine, rationally designed to overcome multiple mechanisms of resistance, inhibit the classical and novel isoforms of protein kinase C, is able to cross the blood brain barrier and is orally bioavailable. KPC34 had an IC_50_ in the nanomolar range against multiple ALL cell lines tested but was lowest for Ph+ lines. In mice bearing either naïve or resistant Ph+ ALL, KPC34 treatment resulted in significantly improved survival compared to cytarabine and gemcitabine. Treatment with KPC34 and doxorubicin was more effective than doxorubicin and cytarabine. Mice with recurrence of their ALL after initial treatment with cytarabine and doxorubicin saw dramatic improvements in hind limb paralysis after treatment with KPC34 demonstrating activity against established CNS disease. Consistent with this KPC34 was better than gemcitabine at reducing CNS leukemic burden. These promising pre-clinical results justify the continued development of KPC34 for the treatment of Ph+ALL.

## Introduction

Ph+ ALL is a cancer of the blood and bone marrow that causes an accumulation of immature lymphoblasts, leading to bone marrow failure and ultimately death [1]. It accounts for approximately 25% of all adult ALL cases and has historically been associated with a poor prognosis (reviewed by Fielding[2]). Outcomes have improved with the advent of TKIs that target the BCR-ABL fusion protein generated by the Philadelphia chromosome but these approaches are not curative in the absence of a stem cell transplant and even with transplant the long term survival is still only 40–50%[3].

In Ph+ ALL, the targeting of BCR-ABL with tyrosine kinase inhibitors (TKIs) results in high initial response rates. However, when TKIs are used as single agents responses are short-lived with rapid development of resistance [4]. This is likely a reflection of the intra-tumoral genetic heterogeneity contained within ALL patients (reviewed in [5]). In both of these examples responses have been made more durable by combining a targeted agent with cytotoxic chemotherapy. Indeed, the combination of a TKI with chemotherapy has now become the standard of care for patients with Ph+ ALL [2]. This has led to remission rates in adults of up to 90 to 100% of patients[6]. However, in patients unable to receive a stem cell transplant remissions are transient and relapsed disease is much more difficult to treat with median survival of less than a year[7]. Central nervous system (CNS) involvement occurs in ~10% of patients at diagnosis and increases to 30% at relapse [8–10] and all patients are treated with CNS prophylaxis most commonly in the form of intrathecal chemotherapy. Patients with documented CNS leukemia require cranial radiation, high-dose chemotherapy and/or intrathecal injections, all of which have undesirable side effects [11].

Nucleoside analogues like cytarabine have long been the backbone of chemotherapy for ALL. However, cytarabine is a prodrug that requires several cellular enzymes for leukemia cell uptake and metabolism before it becomes the active triphosphorylated metabolite, Ara-CTP. It must enter the cell via an equilibrative nucleoside transporter (ENT-1), and needs to be phosphorylated by deoxycytidine kinase (dCK), the rate limiting step for its activation. Consistent with this, down-regulation of ENT-1 and dCK confer a poor prognosis in leukemia and are likely sources of resistance[12, 13].

KPC34 is a first in class, novel phospholipid gemcitabine conjugate, consisting of gemcitabine monophosphate attached to an amido-containing diacylglycerol (DAG) mimetic (S1 Fig). It is rationally designed to simultaneously deliver a Protein Kinase C (PKC) inhibitor and a DNA damaging agent. It is orally bio-available, able to cross the BBB, and taken up independently of cellular nucleoside transport proteins like ENT-1. Once taken up by a leukemia cell it is cleaved by the upstream activator of PKC, phospholipase C (PLC), to generate gemcitabine monophosphate and the DAG mimetic. Since gemcitabine monophosphate is formed by PLC cleavage this bypasses the need for dCK.

Protein kinase C is a family of at least 12 related proteins with diverse cellular functions whose dysregulation has been implicated in oncogenesis [14]. The classic members (PKCα, β1, β2 and γ) require calcium and either diacylglycerol (DAG) or phosphatidylserine (PS) for activity. The novel members (PKCδ, ε, η, and θ) require only DAG or PS for activity. PKC lies immediately downstream of PLC which is in turn activated by multiple key signaling pathways in ALL including BCR-ABL [15–17]. The α and β family members are highly expressed in ALL cell lines and primary patient samples and targeting these kinases is cytotoxic [18]. PKC isoforms have also been implicated in resistance to DNA damaging agents, including cytarabine in ALL cells [19].

In this study we sought to determine the activity of KPC34 against preclinical models of treatment naïve as well as resistant Ph+ ALL.

## Materials and methods

### Reagents

KPC34 was synthesized as previously published in [20]. Gemcitabine Hydrochloride (#G6423) and dipyridamole (#D9766) were purchased from Sigma-Aldrich (St. Louis. MO). Cytarabine was purchased from NOVAPLUS and Doxorubicin HCl was purchased from Pfizer (New York, NY). For animal studies, all chemotherapeutic agents were dissolved in saline prior to treatment.

### Cell culture

The mouse leukemia line B6 ALL was maintained in 45% DMEM, 45% IMDM, and 10% FBS supplemented with L-glutamine, penicillin, and streptomycin. The cell lines CCRF-CEM, MOLT4, and Jurkat were maintained in RPMI media (Gibco, Carlsbad, CA) supplemented with 10% FBS, penicillin and streptomycin. SUPB15 was maintained in IMDM media with 20% FBS, L-glutamine, penicillin, streptomycin and 50 uM 2-mercaptoethanol. Cell lines SUP-B15 and Jurkat were luciferase tagged by lentiviral infection with a luciferase expressing vector by the Cell virus and vector laboratory and subjected to clonal derivation by limiting dilution. The M2 cell line was grown in DMEM with 10% FBS, penicillin, and streptomycin. Cell lines were maintained in an incubator at 37°C and 5% CO_2_.

### Cell viability

Viability assays were performed in 96 well plates using the Cell Titer-Glo assay (Promega, Madison, WI) according to the manufacturer's protocol. Murine cell were plated at 50,000 cells/ml and human cells were plated at 100,000 cells/ml prior to drug exposure. Cells were plated at 500 μl volume in triplicate for each treatment.

### Flow cytometry assays

100,000 cells/ml in 0.5 ml, were grown for 2 days and treated for 48 hours. After washing in cold PBS, cells were stained with propidium iodide (Sigma Aldrich, St. Louis, MO) and allophycocyanin (APC)-conjugated annexin V according to the manufacturer’s protocol. Flow cytometry was conducted on an Accuri C6 cytometer (BD Pharmingen, San Jose, CA) with the FCS Express software (De Novo Software, Los Angeles, CA). For co-culture assays, cells were plated at 50,000 cells/ml alone in 0.5 ml or in 0.25 ml with 0.25 ml of M2 stromal cells at 50,000 cells/ml. The cells were incubated for 48 hours and then treated for 48 hours. Non-adherent cells were collected 96 hours after initial plating and annexin V/propidium iodide staining was performed. Dual staining of γ-H2AX and B220R was performed on bilateral femur cells harvested from mice. Cells from each mouse were stained with: a) Rat anti-mouse B220R FITC Ab (#553087, BD Biosciences, San Jose, CA) b) γ-H2AX S139 Rabbit mAb AlexaFluor 647 (#9720, Cell Signaling, Beverly, MA), c) B220R + γ-H2AX, d) Rat IgG2a k Isotype Control FITC (#553929, BD Biosciences, San Jose, CA), or e) Rabbit IgG Isotype Control Rabbit Ab AlexaFluor 647 (#3452, Cell Signaling, Beverly, MA). Cells were gated by size and B220R positivity.

### Western blotting

Cells were lysed in Laemmli buffer and samples separated by SDS-PAGE before transfer to a PVDF membrane (Millipore, Billerica, MA). Primary antibodies against caspase 3 (#9662 Cell Signaling, Beverly, MA), actin (AC-15, 1:10000; Abcam, Cambridge, MA), anti-pH2AX (#2577, 1:1000; Cell Signaling, Beverly, MA), and phospho-PKCα/β2 (#9375, 1:1000; Cell Signaling, Beverly, MA), and a secondary antibody anti-mouse (#7076, 1:5000; Cell Signaling, Beverly, MA or anti-rabbit (#7074, 1:5000; Cell Signaling, Beverly, MA) were used. ImageJ software (http://imagej.nih.gov/ij/) was used to quantify band intensity.

### Mouse studies

The Comprehensive Cancer Center of Wake Forest University Institutional Animal Care and Use Committee approved all mouse experiments. B6-ALL cells (1x10^6^) were injected into 8 week old C57/Bl6. Luciferase tagged Jurkat or SUP-B15 cells (1x10^6^) were injected into 8 week old NSG-transgenic mice that express human iL3, CSF2, SCF (Jackson Labs strain 013062). Mice were imaged using an IVIS100 system (Caliper LifeSciences, Hopkinton, MA). Mice were injected with 150 mg/kg D-Luciferin (Gold Biotechnology, St. Louis, MO), and imaged for 2 min. Chemotherapy was initiated upon detection of signal. Mice were randomly assigned to treatment groups and treated as described. For combination studies, mice were treated with KPC34 or Ara-C once a day for four days and doxorubicin at 3 mg/kg once a day for three days. For the duration of treatment, drinking water was supplemented with 1 mg/ml Neomycin. Animals were euthanized upon onset of hind limb paralysis, hunched posture, or difficulty breathing. In CNS studies, brains were fixed in 10% neutral-buffered formalin for 72 hours and then transferred to Surgipath Decalifier-I solution (10% v/v formaldehyde-formic acid, Leica, Buffalo Grove, IL) for 96 hours. Samples underwent routine tissue processing and sectioning for H&E staining. Photographs of tissues were taken with the NIS Elements D3.10 camera and software system. Dipyridamole was dissolved in 12.5% EtOH in D5 water (5% dextrose). During the diet study, high-fat diet (D12492, Research Diets) or a calorically equivalent low-fat diet (D12492 Match 7% Sucrose) was given during treatment after which mice were returned to normal chow.

### Ara-C resistant ALL

To generate Ara-C resistant cells, mice were injected with B6-ALL leukemic cells as described above. Mice were treated with 100 mg/kg Ara-C daily until euthanasia was required. Bilateral femur cells were harvested and the cells were cultured in 45% DMEM, 45% IMDM, and 10% FBS supplemented with L-glutamine, penicillin, and streptomycin. Naïve C57/Bl6 mice were then injected with 500,000 cells by tail vein injection. Prior to treatment, leukemic engraftment was confirmed by IVIS imaging.

### Toxicology studies

Normal C57/Bl6 mice were treated with identical dose, schedule, and route of each drug as in the efficacy studies (days 1–4). Seventy-two hours after the last dose, animals were sacrificed and organs were fixed in 10% neutral-buffered formalin followed by routine tissue processing and sectioning for H&E staining. Photographs of tissues were taken with the NIS Elements D3.10 camera and software system.

### Statistical analysis

Groups of 3 or more were analyzed using a one way ANOVA. All means were compared by a student's 2-tailed t test. The *in vivo* survival graphs were generated with the Kaplan-Meier method, with p values determined by the log-rank test. Repeated measures were analyzed using a two way ANOVA All analyses were performed using GraphPad Prism Version 5.02 (GraphPad Software). A p value ≤ 0.05 was considered to be significant.

## Results

### KPC34 induces apoptosis in Ph+ ALL cells

**Fig 1:**
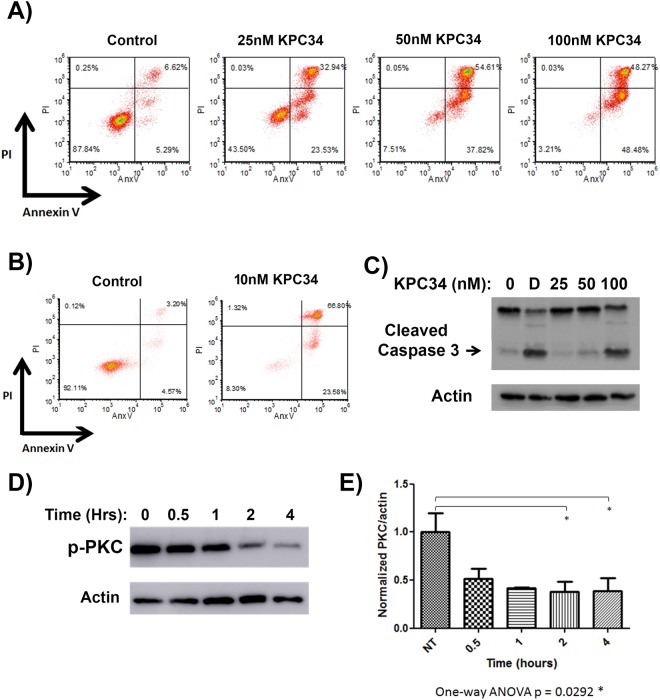
KPC34 induces apoptosis in a dose-dependent fashion and inhibits PKC phosphorylation. A) Flow cytometry of annexin V assay. B6-ALL cells were treated with the indicated amount of KPC34 for 48 hours and then labeled with annexin V and propidium iodide (PI).B) Flow cytometry of annexin V assay. SUPB15 cells were treated with the indicated amount of KPC34 for 48 hours and then labeled with annexin V and propidium iodide (PI).C) Western blot analyses of apoptotic induction. B6-ALL cells were treated with the indicated amount of KPC34 for 48 hours or for four hours with 500 ng/ml doxorubicin (D) as a positive control and blotted for caspase 3. Arrow denotes cleaved caspase 3. Actin was used as a loading control.D) Western blots of phosphorylated PKCα and β_II_. B6-ALL cells were treated with 200 nM KPC34 for the indicated time and were blotted with an antibody that recognizes both phosphorylated PKCα and PKCβ_II_ (p-PKC). Actin was used a loading control.E) Quantified p-PKC expression of B6-ALL cells treated with vehicle or KPC34 200 nM. Band intensities from three independent experiments were calculated using ImageJ software and p-PKC values were normalized to actin for each replicate. Errors bars represent the SEM.

In order to determine the activity of KPC34 against acute lymphoblastic leukemia we determined the IC_50_ values of a panel of human and murine cell lines The IC_50_ values for all lines tested were in the nanomolar range however for both Ph+ cell lines the IC_50_ was twofold or more lower at 10 nM or less (Table 1). Given this extreme sensitivity we focused our study on Ph+ ALL models. To determine if KPC34 was cytostatic or cytotoxic, the murine Ph+ ALL cell line B6-ALL was incubated with 25, 50 or 100 nM for 48 hours and apoptosis assessed by Annexin V/Propidium Iodide (AnxV/PI) staining (Fig 1A). A dose-dependent increase in dual-stained populations was seen, demonstrating induction of apoptosis by KPC34. We also tested KPC34 against the human Ph+ ALL line SUP-B15 and again saw induction of annexin V and PI staining (Fig 1B). Induction of apoptosis was further confirmed by immunoblotting for cleaved caspase 3 in B6-ALL cells treated with KPC34 for 48 hours (Fig 1C). These findings demonstrate that KCP34 induces apoptosis in Ph+ ALL cells in a dose-dependent fashion.

**Table 1:** KPC34 is active against ALL *in vitro* in the nanomolar range.

Cell Line	Properties	KPC34 IC_50_ (95% CI)
**B6-ALL (Ph+)**	Murine B ALL, BCR-ABL, ARF-	10.36 nM (8.448–12.7)
**SUPB15 (Ph+)**	Human B cell ALL, BCR-ABL	7.39 nM (6.25–8.73)
**Jurkat**	Human T cell ALL	27.94 nM (22.88–34.12)
**MOLT4**	Human T cell ALL, heterozygous p53 (point missense mutation)	42.71 nM (32.59–55.96)
**CCRF-CEM**	Human T cell lymphoblastic ALL, compound heterozygote with two p53 point mutations	175.6 nM (146.8–210.0)

### KPC34 inhibits PKC activation

Cleavage of the phospholipid group on KPC34 by phospholipase C is predicted to produce a DAG mimetic capable of inhibiting the conventional and novel PKC isoforms. PKC requires an auto-phosphorylation to become fully active (reviewed in [21]). Since the classic isoforms have been shown to be highly over-expressed in ALL cell lines and patient samples we focused on PKC α and β_II_[18]. To test KPC34’s ability to inhibit their auto-phosphorylation, B6-ALL cells were incubated with KPC34 and cell lysates collected for Western blotting with an antibody specific for both phosphorylated PKC α (at Thr638) or PKC β_II_ (at Thr641). A decrease in phosphorylated PKC α/β_II_ in cells treated with KPC34 was observed (Fig 1D). A statistically significant reduction in PKC phosphorylation was seen between the vehicle-treated cells and cells treated with KPC34 (p = 0.0292 by one-way ANOVA, Fig 1E). These findings demonstrate that KPC34 inhibits the auto-phosphorylation of PKC needed for its activation.

### KPC34-induces DNA damage in vivo

**Fig 2:**
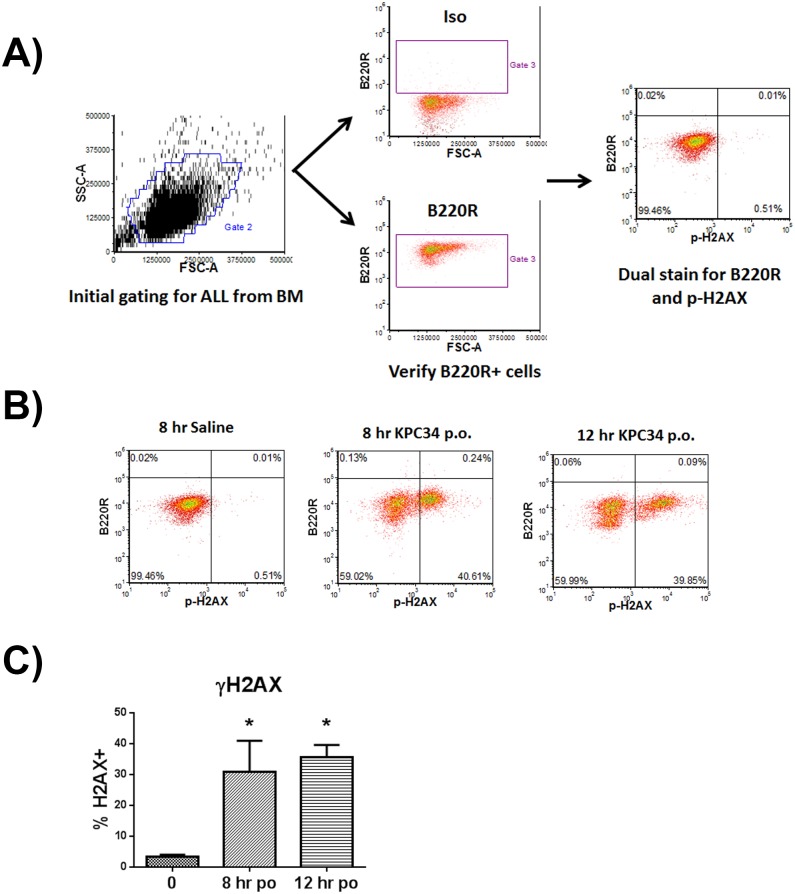
Oral administration of KPC34 induces DNA damage *in vivo*. A) Harvested cells were stained with antibodies for B220R and H2AX and analyzed by flow cytometry. Cells were initially gated by size, followed by B220R positivity. Dual-stained populations were then measured after gating saline treated cells.B) Flow cytometry analyses of harvested bone marrow cells stained with B220R and H2AX. Representative dot plots are shown.C) Quantified dual-stained populations averaged from 3 independent replicates. Values represent Mean + SEM. Tukey’s post t test was performed following one-way ANOVA. * = p value <0.05.

*In vitro* cytotoxicity assays do not recapitulate the important contributions of pharmacokinetics, immune system interactions and the native microenvironment seen in patients. In order to assess if orally administered KPC34 at tolerable doses is delivered to the bone marrow at active concentrations we utilized a syngeneic, immunocompetent, orthotopic mouse model of Ph+ pre-B cell ALL (B6 All) [22]. Using this model we first determined the maximally tolerated dose in mice when given daily for up to 4 doses. C57/Bl6 mice were able to tolerate doses of 20 mg/kg given orally for up to 4 doses with transient weight loss only (data not shown). We then tested the effect of this dose of KPC34 on DNA damage induction by γ-H2AX staining. Mice were tail vein injected with 1x10^6^ B6 ALL cells and upon leukemic engraftment, were treated with saline or KPC34 orally and sacrificed 8 or 12 hours later. Bone marrow was harvested from femurs and stained for the pan B cell marker B220R (expressed by B6 ALL cells), γ-H2AX or both. Cells were analyzed by flow cytometry and leukemic cells were gated by size and B220R positivity (Fig 2A). Induction of γ-H2AX was analyzed in B220R+ cell populations (Fig 2B). γ-H2AX induction was significantly increased at both time points compared to controls (Fig 2C). These findings demonstrate that KPC34 20 mg/kg given orally effectively induces DNA damage in leukemia cells within the bone marrow.

### KPC34 is efficacious as a single agent against untreated Ph+ ALL *in vivo*

**Fig 3:**
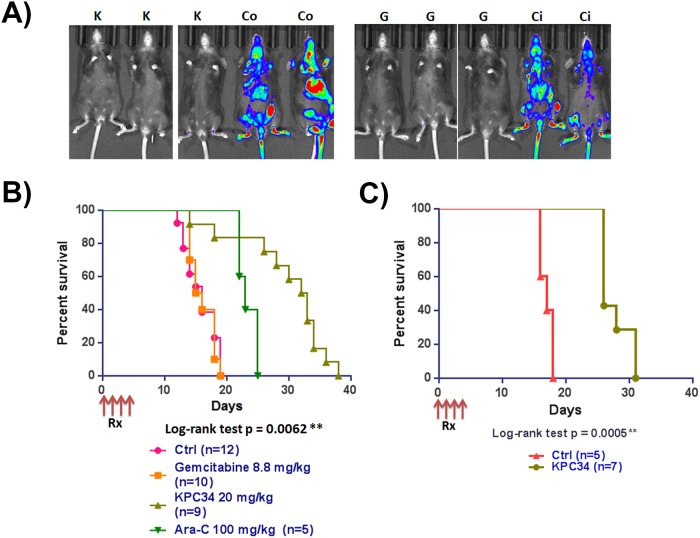
KPC34 is active *in vivo* against a syngeneic model of murine ALL. A) C57/Bl6 mice were injected with B6-ALL syngeneic leukemia. Upon engraftment mice were treated with saline p.o. (Co), saline i.p. (Ci), Gemcitabine at 8.8 mg/kg i.p. (G), or KPC34 at 20 mg/kg p.o. (K). Bioluminescent image of mice 24 hours after the last treatment with saline, gemcitabine or KPC34.B) Kaplan-Meier curves for C57Bl/6 mice treated with saline, gemcitabine, cytarabine or KPC34 as indicated.C) Kaplan-Meier curves for Transgenic NSG tail vein injected with SUPB15 cells and treated with saline (Ctrl) or KPC34 at 10 mg/kg p.o.

*In vitro* cytotoxicity assays and demonstration of induced DNA damage do not establish efficacy. In order to assess efficacy mice were tail vein injected with B6-ALL cells and engraftment was confirmed six days later by bioluminescent imaging. Upon confirmation of engraftment mice were randomly assigned to receive either vehicle control (PBS), 20 mg/kg of KPC34 by oral gavage, equimolar (8.8 mg/kg) gemcitabine intraperitoneal (IP) or the maximally tolerated dose of cytarabine (100 mg/kg) IP once daily for four days. Although not used in the treatment of ALL, equimolar gemcitabine was included in the experiment to show that the effect of KPC34 was not simply due to delivery of the gemcitabine moiety in the molecule. Cytarabine is commonly used in consolidation and/or late intensification in the treatment of ALL [23, 24] and was included to show the effect of a more traditional nucleoside analog on our model. Following treatment there was a dramatic reduction in total leukemic burden in treated mice by bioluminescent imaging (Fig 3A) compared to controls. Despite this, gemcitabine treatment provided little survival benefit with only a one day increase in median survival compared to controls. In contrast, KPC34 treatment resulted in a significant survival advantage (p = 0.0001) compared to any other treatment group (control, gemcitabine or cytarabine), with a median survival of 32 days compared to 14.5, 15.5 or 23 respectively (Fig 3B). To extend these results to human derived models we tail vein injected luciferase tagged SUP-B15 cells into NSG-transgenic mice. Upon confirmation of engraftment animals were randomly assigned to KPC34 10 mg/kg daily x4 (the maximally tolerated dose in NSG-transgenic mice) or PBS control treatments and followed for survival. Consistent with the murine model, KPC34 treatment resulted in a significant survival benefit (p = 0.0005) with control animals having a median survival of 17 days compared to 26 days for KPC34 treated (Fig 3C). These data demonstrate that KPC34 given orally is active against both murine and human Ph+ ALL models. Further, KPC34 was more efficacious against the murine model than equimolar gemcitabine or the maximally tolerated dose of cytarabine.

### KPC34 reduces CNS burden in mice bearing Ph+ ALL

**Fig 4:**
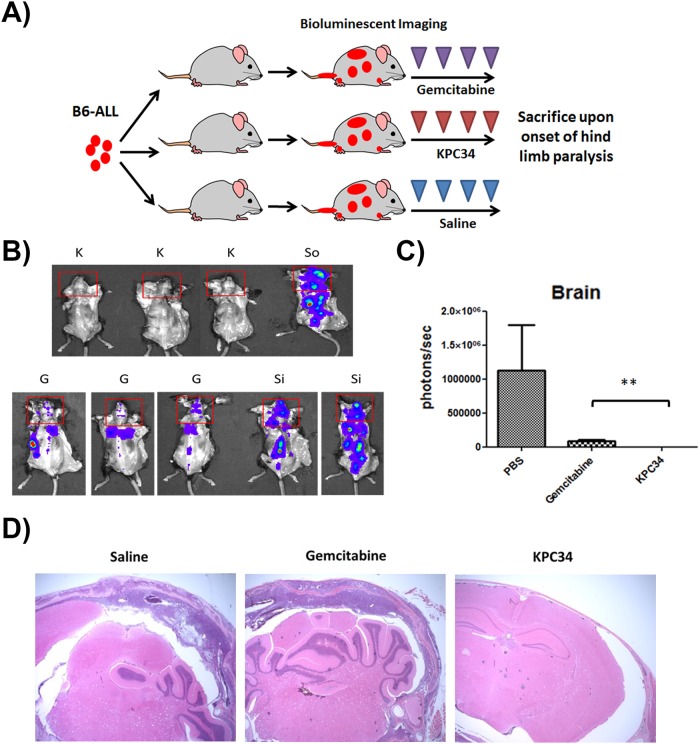
KPC34 reduces CNS burden in mice bearing ALL. A) Schema of treatment trial. C57/Bl6 mice were tail vein injected with B6-ALL cells. Upon engraftment, mice were treated with saline p.o. (So), saline i.p. (Si), Gemcitabine at 8.8 mg/kg i.p. (G), or KPC34 at 20 mg/kg p.o. (K). Upon onset of hind limb paralysis in control mice, all mice were imaged and euthanized.B) Bioluminescent image of mice on Day 14 from injection. Red squares were used to gate leukemic infiltration within the brain.C) Quantified total luminescence in brains of treated mice on Day 14. Values represent Mean + SEM. Unpaired t test was performed following one-way ANOVA. ** = p value ≤ 0.01.D) Representative slides of fixed, sectioned and H+E stained brains of mice treated as indicated.

A major trigger for euthanasia in our syngeneic, orthotopic mouse model is hind limb paralysis secondary to CNS infiltration of leukemia. Given its hydrophobic nature, KPC34 is predicted to cross the blood brain barrier. As we observed a dramatic increase in survival with KPC34 over gemcitabine despite little difference in total leukemic burden by bioluminescent imaging (Fig 3A and 3B) we hypothesized that the difference was due to reduced or delayed leukemic infiltration of the CNS. To test this hypothesis we tail vein injected B6-ALL cells and treated mice as in Fig 3. However in this experiment all mice were sacrificed upon the onset of hind limb paralysis in control treated animals (Fig 4A). Mice were injected with luciferin, sacrificed 5 minutes later, had the skin resected and were imaged. Brains were exposed during dorsal imaging. Total luminescence was quantified within the brain (Fig 4B and 4C). Significant differences were found between mice treated with gemcitabine or KPC34 (Fig 4B and 4C). CNS infiltration was also assessed by H+E staining of the harvested brains (Fig 4D). A veterinary pathologist blinded to treatment observed CNS involvement in saline treated mice and to a lesser extent in gemcitabine treated mice. In all mice treated with KPC34, no CNS leukemic infiltration was observed. These studies show that KPC34 reduces CNS infiltration by leukemic cells more effectively than gemcitabine.

### KPC34 can be combined with nilotinib against Ph+ ALL *in vivo*

**Fig 5:**
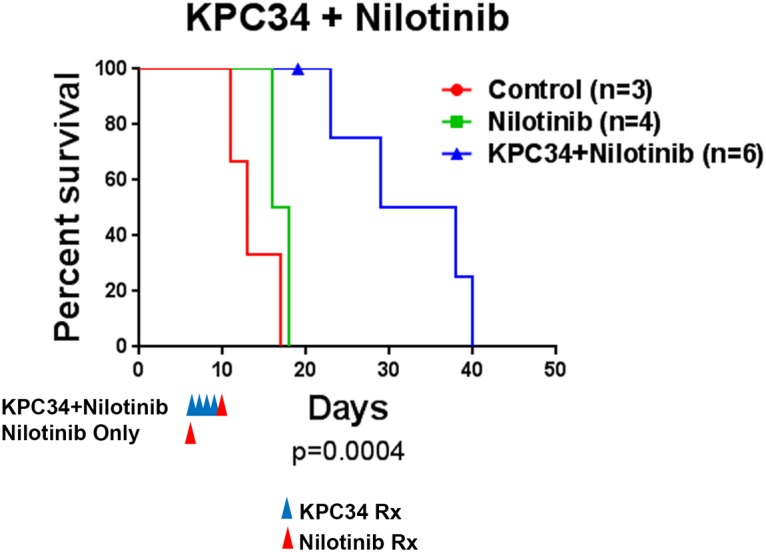
KPC34 can be combined with a TKI. Kaplan-Meier curves for C57/Bl6 mice injected with B6-ALL syngeneic leukemia. Upon engraftment mice were treated with saline (control), nilotinib at 75 mg/kg or KPC34 at 20 mg/kg daily for 4 days followed by nilotinib at 75 mg/kg. The p value shown was calculated by log-rank test.

The advent of tyrosine kinase inhibitors (TKIs) has revolutionized the treatment of Ph+ ALL patients and these medications are now part of the standard of care. The tyrosine kinase inhibitor nilotinib has been used in the treatment of Ph+ B cell ALL in the relapsed and up-front settings [25, 26]. In order to see if TKI therapy could be combined with KPC34 we tail vein injected B6-ALL cells into C57Bl/6 mice and upon engraftment (day 6 from injection) animals were randomly assigned to treatment. Mice were treated with saline (control) or KPC34 at 20 mg/kg daily followed by nilotinib 75 mg/kg on day 5 or nilotinib treatment on day one followed by 4 days of saline. We chose to treat the nilotinib only group on day one so to ensure similar disease burden at initiation of therapy with the KPC34 and control groups. Nilotinib as a single dose provided a small survival benefit that appeared additive when combined with KPC34 (Fig 5). These data suggest that TKI therapy can be combined with KPC34.

### KPC34 is efficacious in combination with doxorubicin against Ph+ ALL *in vivo*

**Fig 6:**
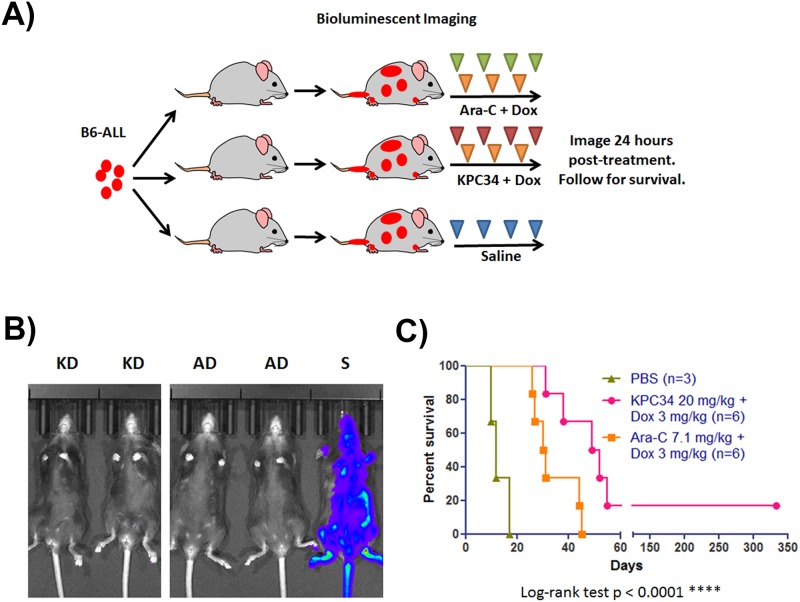
KPC34 is more effective than Ara-C when combined with doxorubicin. A) Schema of treatment trial. C57/Bl6 mice were injected with B6-ALL syngeneic leukemia. Upon engraftment as confirmed by bioluminescent imaging, mice were treated with saline by oral gavage and i.p. (S), Ara-C at 7.1 mg/kg i.p. for four days and Dox at 3 mg/kg i.p. for three days (AD), or KPC34 at 20 mg/kg p.o. for four days and Dox at 3 mg/kg i.p. for three days (KD).B) Bioluminescent image of mice 24 hours after the final treatment.C) Kaplan-Meier curves for animals treated with saline, Ara-C + Dox, or KPC34 + Dox.

The current treatment of ALL involves simultaneous use of multiple chemotherapies throughout all phases of treatment. To evaluate the possibility of using KPC34 as part of a combination regimen, B6-ALL cells were tail vein injected into C57Bl/6 animals as above. Once engraftment was confirmed, mice were treated with doxorubicin for three days and either cytarabine or KPC34 for four days (Fig 6A). Combination treatment with KPC34 significantly increased median survival (p < 0.0001) by over 13 days, with one mouse that survived more than a year after therapy and was euthanized for an unrelated stress induced dermatitis (Fig 6B and 6C). There were no long term survivors in the cytarabine treated cohort. These studies suggest that KPC34 is more efficacious than cytarabine as part of a combination treatment against Ph+ ALL. While the one long term survivor in the KPC34 treated group is intriguing, additional studies will be needed to determine the ability of this combination to produce long term remissions in this model.

### KPC34 treats relapsed CNS disease

**Fig 7:**
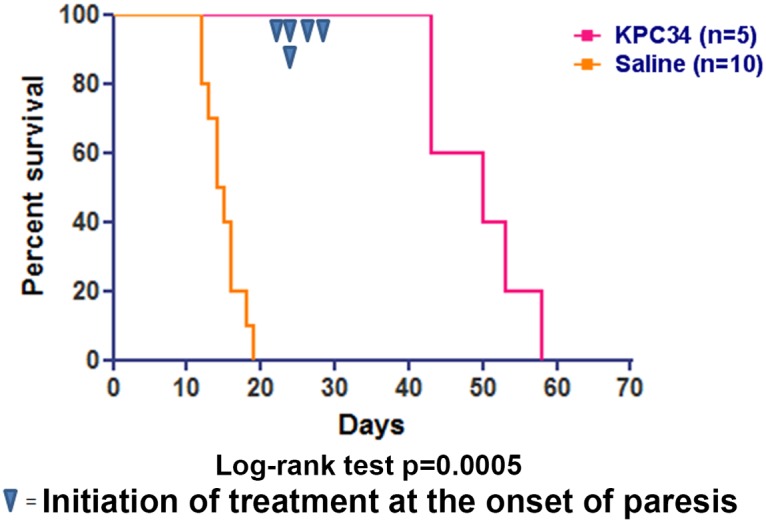
KPC34 is more effective against established CNS leukemia. Kaplan-Meier curves for C57/Bl6 mice injected with B6-ALL syngeneic leukemia. Upon engraftment mice were treated with Ara-C at 7.1 mg/kg i.p. for four days and Dox at 3 mg/kg i.p. for three days. At the onset of hind limb paresis mice were treated with KPC34 at 20 mg/kg p.o. for 2 two days and followed. Once paresis returned mice were treated again.

To assess the ability of KPC34 to treat established CNS leukemia in a relapse setting, we initially treated B6-ALL leukemia-bearing mice (injected and imaged as above) with cytarabine and doxorubicin and then re-treated mice with KPC34 upon onset of hind limb paresis. Mice were treated with two daily doses of KPC34 and then monitored for recurrent hind limb paresis and retreated again. Re-treatment with KPC34 repeatedly reversed hind limb paresis in 24–48 hours and was well-tolerated by mice (see S1 Video). Mice were successfully re-treated multiple times, with one mouse receiving a total of 13 treatments of KCP34 prior to succumbing to disease. The median survival of the retreated mice was significantly longer than those treated with cytarabine and doxorubicin alone (median survival 30.5 vs 50 days, Fig 7). These data demonstrate that KPC34 is tolerated by mice that have been previously treated with chemotherapy and show it has activity in the setting of relapsed leukemia and established CNS disease.

### KPC34 overcomes cytarabine resistance in vivo

**Fig 8:**
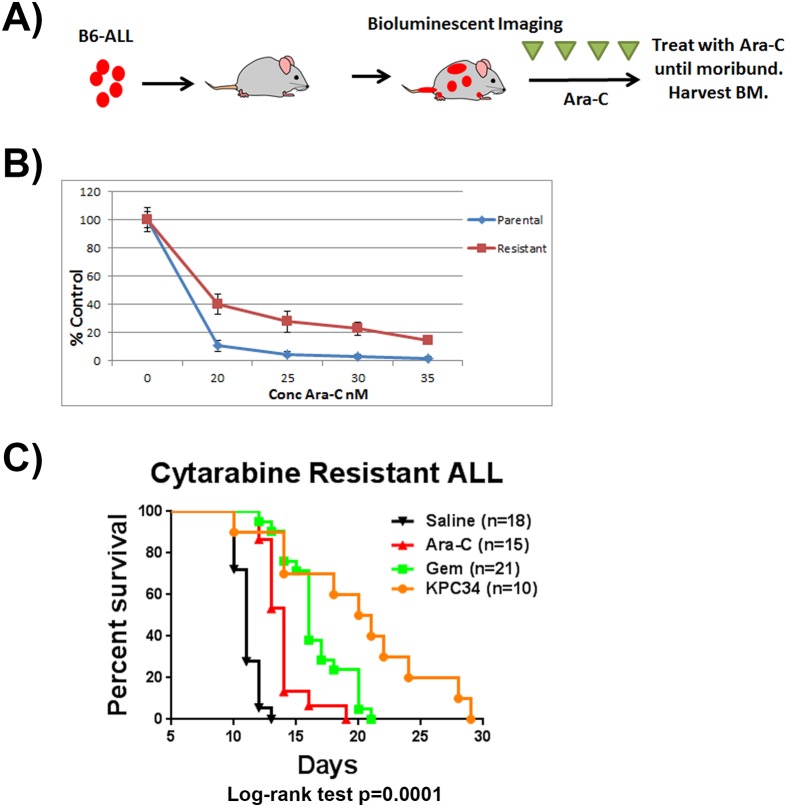
KPC34 is active against an Ara-C resistant model of syngeneic murine ALL. A) C57/Bl6 mice were injected with B6-ALL leukemia. Upon engraftment mice were treated with 100 mg/kg Ara-C until succumbing to disease. Mice were sacrificed and bilateral femur cells were harvested.B) Viability assays of cells harvested from treated mice compared to parental cells. The indicated cells were treated for 72 hours with Ara-C and viability determined.C) Kaplan-Meier curves for animals injected with Ara-C resistant B6-ALL cells. Upon engraftment mice were treated with saline p.o. or i.p., Ara-C at 7.1 mg/kg i.p. (Ara-C), Gemcitabine at 8.8 mg/kg i.p. (Gem), KPC34 at 20 mg/kg p.o. (KPC34).

We have shown that KPC34 is effective against recurrent disease previously treated with cytarabine and doxorubicin however it is not known to what extent that recurrent disease is truly resistant to nucleoside analog therapy. To test the ability of KPC34 to overcome disease that has been repeatedly treated with cytarabine *in vivo*, we generated a cytarabine exposed model (Fig 8A). To create this model we tail vein injected C57Bl/6 mice with B6-ALL cells, and upon disease engraftment repeatedly treated with cytarabine until they succumbed to disease. Leukemic cells were harvested from bilateral femurs and briefly cultured *in vitro*. Resistance to cytarabine was confirmed (Fig 8B). Cells were then injected into naïve secondary recipients. Upon engraftment, mice were treated with saline, or equimolar doses of cytarabine IP, gemcitabine IP, or KPC34 orally. KPC34 treatment significantly increased survival compared to all other treatments (p < 0.0001, Fig 8C). These data suggest that KPC34 remains active in the treatment of disease that has been extensively treated with cytarabine.

### KPC34 is still efficacious in TKI resistant models of Ph+ B cell ALL

**Fig 9:**
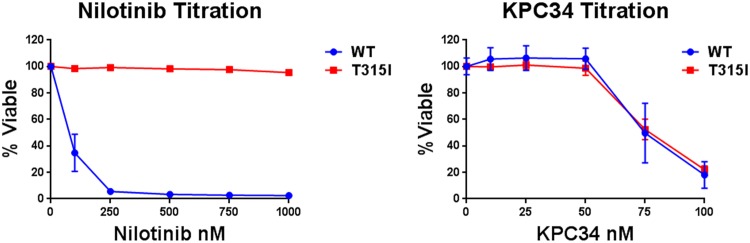
Nilotinib resistant T315I mutant cells are still sensitive to KPC34. Baf-3 cells expressing either wild type (WT) or T315I mutant BCR-ABL (T315I) were treated with the indicated amount of nilotinib or KPC3 for 72 hours and viability assessed. Shown are the averages of 3 independent experiments each done in triplicate.

While TKI therapy has revolutionized the care of Ph+ B cell ALL patients these agents are not cures for the disease and when used as single agents relapse is common. Relapsed disease frequently harbors BCR-ABL kinase domain mutations that block TKI but still allow for substrate binding rendering the TKI ineffective. The most common of these is the T315I mutation that renders BCR-ABL resistant to all TKIs except ponatinib [27]. To see if KPC34 would be active in the setting of a T315I mutation we infected Baf-3 cells with viral vectors that express either wild type (WT) or T315I mutated BCR-ABL and assessed their sensitivity to nilotinib and KPC34. As predicted WT expressing cells were sensitive to nilotinib while T315I expressing cells were not. In contrast both cell lines were sensitive to KPC34 with essentially overlapping growth inhibition curves (Fig 9). These data suggest that ABL kinase mutated Ph+ B cell ALL cells while resistant to nilotinib retain sensitivity to KPC34.

### KPC34 overcomes in vivo inhibition of ENT1

**Fig 10:**
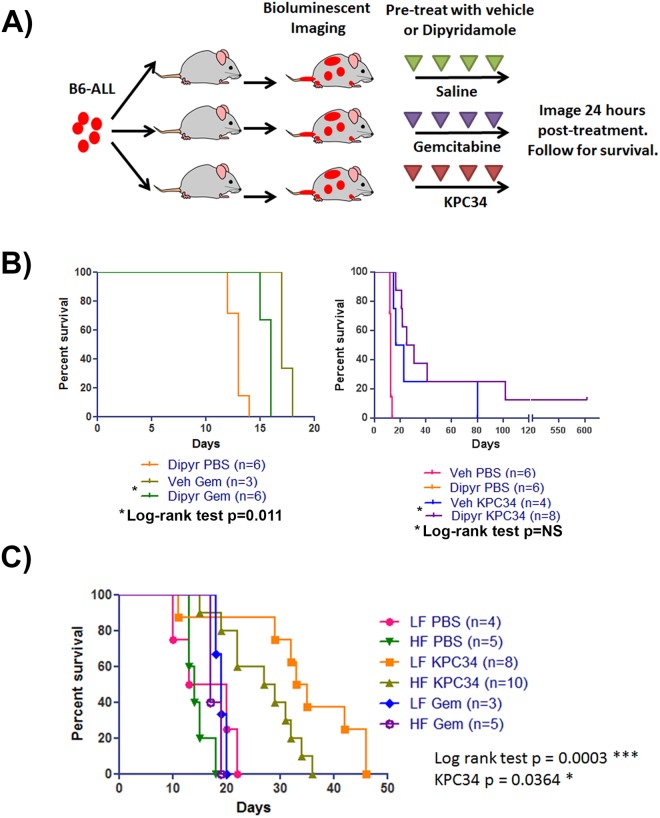
KPC34 overcomes *in vivo* inhibition of ENT1 and dietary fatty acids compete with its uptake. A) Schema of treatment trial. C57/Bl6 mice were injected with B6-ALL syngeneic leukemia. Upon engraftment mice were divided into two groups. One hour prior to receiving chemotherapy, one group was pre-treated with 200 mg/kg Dipyridamole p.o. (D) while the other group was pre-treated with vehicle (V). Mice were then treated with saline (PBS), Gemcitabine at 8.8 mg/kg i.p. (Gem), or KPC34 at 20 mg/kg p.o. (KPC34).B) Kaplan-Meier curves for animals treated with saline, Gemcitabine, or KPC34 pre-treated with vehicle or Dipyridamole. NS = not significant.C) Kaplan-Meier curves for animals treated with saline (PBS), Gemcitabine (Gem), or KPC34 (KPC34) while on a low-fat or high-fat diet. LF = low fat, HF = high fat.

In contrast to cytarabine and gemcitabine, KPC34 is predicted to enter cells by transporter independent uptake. This was assessed by inhibition of the major nucleoside transporter, ENT1, *in vivo*. Gemcitabine and Ara-C require ENT1 to cross the cell membrane and induce cell death. To assess this hypothesis leukemic mice were treated with the ENT1 inhibitor dipyridamole or vehicle one hour prior to treatment with either gemcitabine or KPC34 (Fig 10A). As predicted dipyridamole treatment suppressed gemcitabine uptake and significantly reduced median survival in gemcitabine-treated mice (p = 0.0111 by log rank test). In contrast, no significant difference in median survival was seen in KPC34-treated mice (Fig 10B). Surprisingly, KPC34 treated mice pre-treated with dipyridamole had a numerical increase in median survival compared to KPC34 with vehicle, although the difference was not significant. This may be the result of decreased cytidine transport into leukemia cells in the presence of dipyridamole and may represent a novel additional therapeutic strategy that can be used for nucleoside analogs that do not require ENT1 mediated transport. These data demonstrate that in contrast to the parental drug gemcitabine, the effect of KPC34 is not diminished in the presence of ENT-1 inhibition *in vivo*.

### Dietary fatty acids compete with KPC34 uptake in vivo

We hypothesized that the phospholipid mimicking structure of KPC34 may cause it to be packaged into lipoproteins in the GI tract. If this were true then increased dietary fatty acids should compete with KPC34 for uptake resulting in reduced efficacy. To test this idea, we tail vein injected C57Bl/6 mice with B6-ALL cells as above. Once engraftment was confirmed mice were treated with gemcitabine or KPC34 and randomly assigned to either a high-fat (60% kcal, 18.5% C16 palmitic acid) or calorically equivalent low-fat (10% kcal) diet during the four days of treatment. We found a 6 day decrease in median overall survival in KPC34-treated mice that were placed on the high-fat diet compared to treated mice placed on a low-fat diet, and this difference was statistically significant (Fig 10C). The difference in diet did not have a significant effect on mice treated with saline or gemcitabine indicating that the effect was specific to KPC34. These data suggest that dietary fatty acids attenuate the efficacy of KPC34 perhaps via competition for uptake from the GI tract.

### KPC34 has similar toxicity to gemcitabine

Oral administration of KPC34 was tolerated in various mouse models of Ph+ ALL. To test potential toxicity, healthy C57Bl/6 mice were treated once a day for four days with saline, KPC34 orally or equimolar doses of gemcitabine i.p. as above. Three days after the last treatment, mice were euthanized and sterna, spleens, livers, pancreas, intestines, and kidneys were harvested, sectioned and stained. Mice treated with KPC34 displayed mild inflammation in the intestine with some neutrophilic infiltration, occasional autolysis in the spleen and pancreas, and depletion of various hematopoietic precursor cells. Gemcitabine-treated mice displayed similar findings in the spleen, and both groups of treated mice displayed autolysis in the intestine (S2 Fig). The increased GI toxicity observed in mice treated with KPC34 may be due to increased drug exposure *in vivo* from oral administration or higher GI levels of KPC34 (unpublished data, [20]). These data demonstrate that the phospholipid conjugation and PKC inhibition of KPC34 did not induce additional toxicity compared to the parent drug gemcitabine.

## Discussion

Treatment of Ph+ ALL now includes both TKIs and cytotoxic chemotherapy to achieve the best disease control. Despite the high efficacy of this approach in achieving hematologic remission, relapse with TKI resistant disease remains a major clinical obstacle especially in patients not eligible for stem cell transplant [28]. Here we describe the activity of the novel phospholipid mimetic co-drug, KPC34, against preclinical models of Ph+ ALL. KPC34 was cytotoxic against a panel of ALL cell lines with IC_50_s in the nano-molar range but was particularly effective against the two Ph+ ALL cell lines. KPC34 when internalized by cells is converted to its two active moieties by the action of PLC. BCR-ABL, the product of the Philadelphia chromosome, has been shown to cause strong activation of PLC-gamma1 [29] and this may explain in part the particular sensitivity of Ph+ ALL cells. Additionally, PKC provides anti-apoptotic signaling by activation of the NF-κB and Bcl2 signaling axis [19] and lies downstream of BCR-ABL. A recent study indicated that PKC inhibition in BCR-ABL expressing cells was synergistic with tyrosine kinase inhibition [17]. PKC inhibition via the DAG mimetic moiety of KPC34 combined with the induction of DNA damage by the gemcitabine monophosphate moiety may further explain this hypersensitivity.

In this work we mainly utilized a syngeneic, immunocompetent, orthotopic mouse ALL model that closely mimics human Ph+ ALL. We purposefully chose this model as it is an immunocompetent model that does not require even transient immune suppression and cells engraft in the bone marrow and spleen as seen in patients [22]. Mice treated with KPC34 demonstrated significantly longer survival than those treated with gemcitabine or cytarabine even when used at the maximally tolerated dose, thought to mimic high dose cytarabine treatment in patients [30]. The beneficial effect of KPC34 was not limited to murine derived ALL as there was also a significant survival advantage in an orthotopic human derived Ph+ ALL model. In addition, KPC34 was more effective than cytarabine when combined with doxorubicin. In that experiment there was one long term remitter leading to the possibility that KPC34 combined with doxorubicin may result in long term remissions in this model however this will need to be confirmed in larger studies.

CNS involvement of ALL is seen in 10% of patients at diagnosis and up to 30% at relapse [10] and all patients are treated with CNS prophylaxis usually by intrathecal injection of chemotherapy. While dasatinib does cross the blood brain barrier relapse with BCR-ABL kinase domain mutations remains a problem [31]. The ability to treat TKI resistant Ph+ CNS leukemia with an oral agent would represent a major step forward. In animals previously treated with doxorubicin and cytarabine, re-treatment with KPC34 reversed hind limb paresis multiple times demonstrating its efficacy against established CNS leukemia. In addition, Baf-3 cells engineered to express the T315I ABL kinase domain mutation, the most common mutation found in patients that have failed a TKI, did not display any resistance to KPC34 suggesting the lack of cross resistance one would expect given the disparate mechanisms of action.

Prognosis for relapsed ALL in both adults and children is very poor with one report showing only a 7% salvage rate [32]. One major contributor to this dismal outcome is the fact that the initial treatment of the disease involves essentially all known active agents. Mutations that confer resistance to nucleoside analogs are enriched in relapsed disease [33, 34]. Agents like KPC34 that are not susceptible to these mechanisms of resistance would be invaluable additions to the clinical armamentarium. KPC34 was more effective than cytarabine or gemcitabine in treating an ALL model that was previously treated with cytarabine. Though the in vitro resistance of his model was modest this may have been a result of the artificial culture conditions favoring a more sensitive sub-clone allowing its outgrowth. It is worth noting that cytarabine treatment extended survival by seven days in the treatment naïve model in contrast to the three day survival benefit seen with the cytarabine treated model. This benefit suggests KPC34 can overcome mechanisms of resistance to nucleoside analogs however additional studies with genetically defined resistance mechanisms are needed to confirm this conclusion.

Inhibition of ENT1 by dipyridamole significantly reduced survival with gemcitabine but numerically increased survival (although not statistically significant) in mice treated with KPC34 with one long term remitter. In the presence of dipyridamole, leukemia cells would be impaired in their ability to import cytidine and its precursors to compete with KPC34. This may represent an additional novel strategy to maximize its anti-leukemic activity. Increased dietary fat significantly reduced the efficacy of KPC34 but not gemcitabine. This suggests a competition between dietary fat and KPC34 for uptake from the GI tract. It is possible that KPC34 is packed into lipoprotein complexes. Indeed, it may be that the efficacy of KPC34 reflects enhanced uptake by ALL cells via import of drug laden lipoproteins. ALL blasts vigorously take up lipoproteins [35] and serum lipoprotein levels are lower in patients with acute leukemia and return to normal at time of remission [35, 36]. Additionally, studies have shown that lipoproteins can cross the blood brain barrier and such packaging may provide access to CNS involved leukemia [37, 38].

KPC34 was well tolerated with toxicity observed in the intestines, bone marrow, spleen, and pancreas comparable to gemcitabine. This suggests that there is no increase in toxicity to normal tissues with dual DNA damage and PKC inhibition. The mild increase in GI toxicity may reflect the role for PKC signaling in tissue repair after toxin exposure [39]. Importantly, the dose and schedule of KPC34 used in these studies was tolerable by the mice even when repeatedly administered and resulted in survival benefits in both naïve and resistant ALL models. This suggests an acceptable therapeutic window.

Overall, our findings demonstrate that the novel co-drug KPC34 is highly active as a single or combined therapeutic for the treatment of both naïve and resistant Ph+ ALL models, including CNS disease however several questions remain. Additional efficacy studies with patient derived xenograft models would be informative however the rarity of this disease made such models unavailable to us. Mechanistic details of how KPC34 is handled by the GI tract and whether it is packaged into lipoproteins remains unclear. Further study is needed to better establish the individual contributions of PKC inhibition versus DNA damage to leukemia cell killing. It is also unclear to what extent activated PKC predicts efficacy of this approach or whether activity of PLC might be a better predictor. Such investigations will require additional pharmacological inhibitors and/or genetic manipulations and are currently underway. Finally, while KPC34 was most active against Ph+ ALL cell lines there was nanomolar activity against other ALL lines including T cell ALL models suggesting it could be effective in T cell disease as well. This will require additional testing. Despite these limitations we believe that the data justify the continued development of KPC34 for the treatment of Ph+ ALL.

## Supporting information

S1 Fig Chemical structure of KPC34. (TIF)

S2 Fig KPC34 is tolerated by mice *in vivo*. Images of organs from mice treated with gemcitabine or KCP34. Mice were treated with saline (S) or equimolar doses of gemcitabine i.p. (G) or KPC34 (K) by oral gavage for 4 days. 72 hours after the last treatment, mice were sacrificed and the following organs were harvested: sterna, liver, kidneys, spleen, small and large intestines, and pancreases. Samples were processed and stained with hematoxylin and eosin. A blinded veterinary pathologist analyzed the samples. All images are taken at 20X zoom, except for bone marrow taken at 60X.
(TIF)

S1 Video KPC34 reverses hind limb paralysis. Video of a C57Bl/6 mouse injected with B6 ALL cells and previously treated with Ara-C and doxorubicin at the onset of hind limb paralysis prior to KPC34 treatment, 24 hours later after 1 treatment and 48 hours later after 2 treatments.
(MP4)
