# Supplementary figures and images for: The novel phospholipid mimetic KPC34 is highly active against preclinical models of Philadelphia chromosome positive acute lymphoblastic leukemia
Source: PLoS One. 2017 Jun 23;12(6):e0179798. doi: 10.1371/journal.pone.0179798 (PMC5482463; doi:10.1371/journal.pone.0179798)

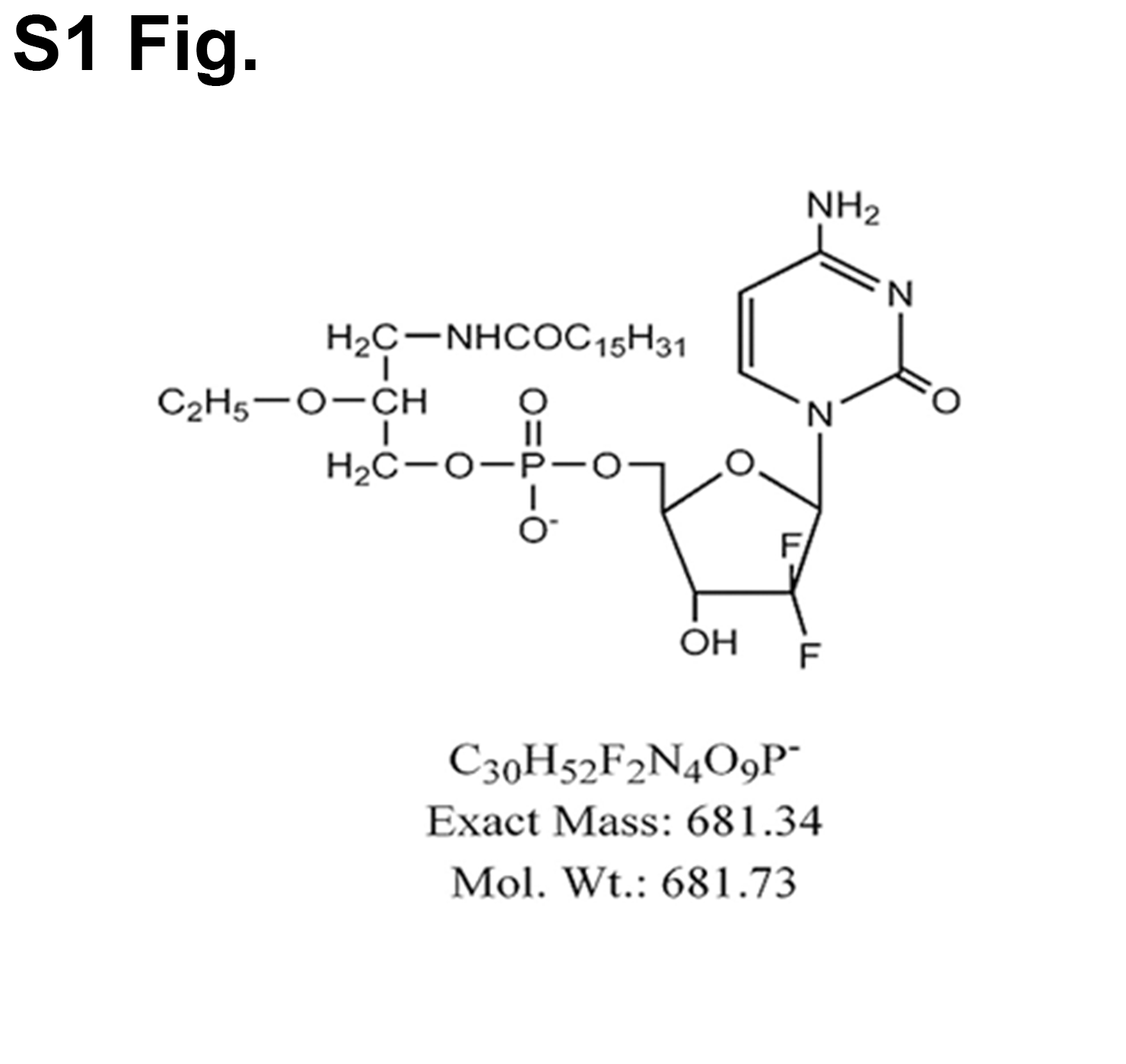

Supplement: S1 Fig — (TIF) [file pone.0179798.s001.tif]

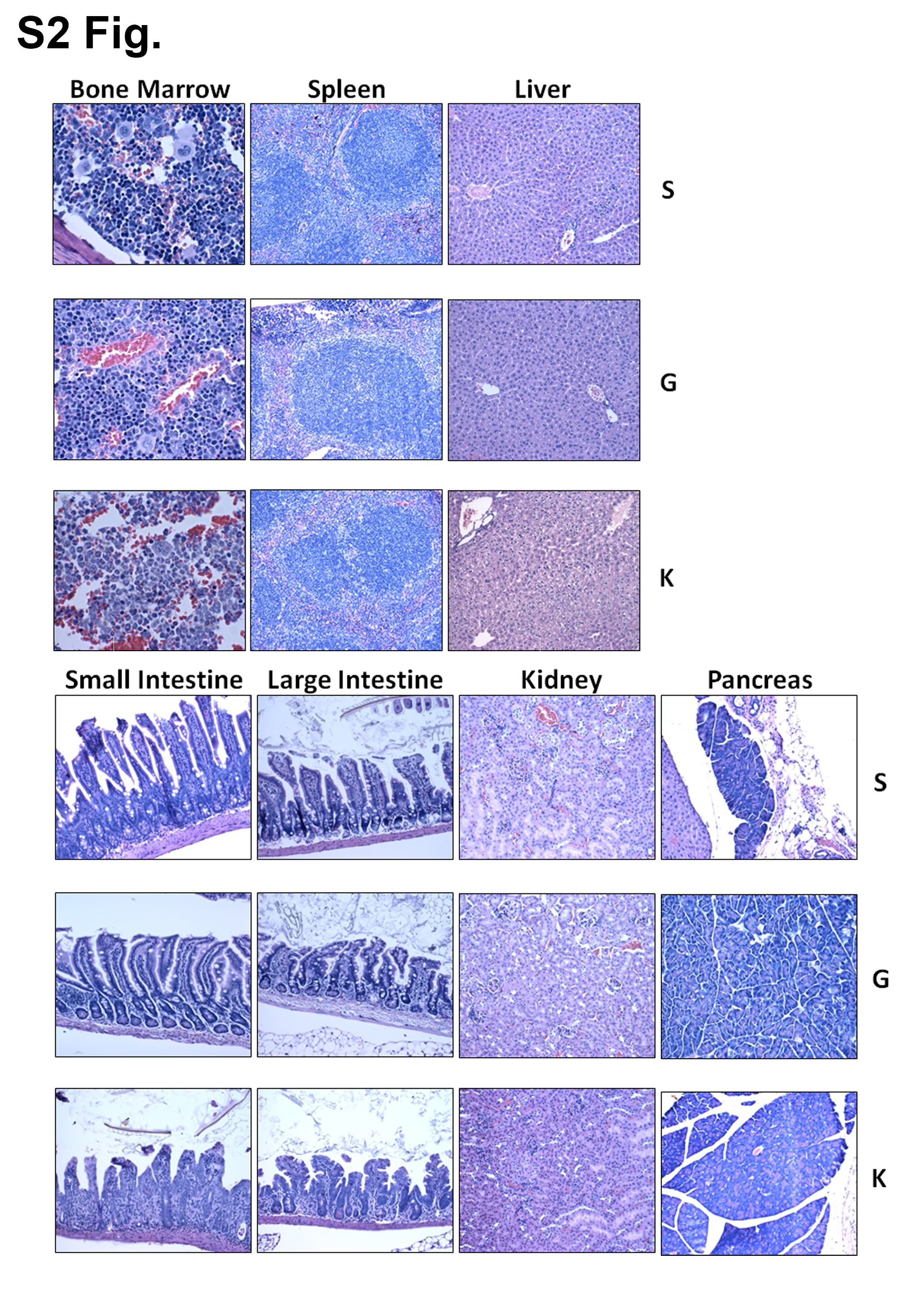

Supplement: S2 Fig — Images of organs from mice treated with gemcitabine or KCP34. Mice were treated with saline (S) or equimolar doses of gemcitabine i.p. (G) or KPC34 (K) by oral gavage for 4 days. 72 hours after the last treatment, mice were sacrificed and the following organs were harvested: sterna, liver, kidneys, spleen, small and large intestines, and pancreases. Samples were processed and stained with hematoxylin and eosin. A blinded veterinary pathologist analyzed the samples. All images are taken at 20X zoom, except for bone marrow taken at 60X. (TIF) [file pone.0179798.s002.tif]
